# S12-1: Can a human rights-based approach help to promote physical activity for vulnerable groups?

**DOI:** 10.1093/eurpub/ckae114.251

**Published:** 2024-09-26

**Authors:** Sven Messing, Karim Abu-Omar, Peter Gelius

**Affiliations:** FAU Erlangen, Department of Sport Science and Sport, Erlangen, Germany; University of Limerick, Ireland; FAU Erlangen, Department of Sport Science and Sport, Erlangen, Germany; Université de Lausanne, Institute of Sport Sciences, Switzerland

## Abstract

**Purpose:**

As physical activity was limited in some countries during the pandemic-related lockdowns, the question whether humans have a right to be active was discussed in public and academia. A recent scientific study has shown how a right to physical activity can be derived from well-established human rights such as the right to health, the right to rest and leisure, the right to education, and the principle of nondiscrimination. However, this is a new perspective for the field of PA promotion, and examples of studies using a human rights-based approach systematically are rare.

This study aimed to develop a framework for utilizing a human rights-based approach in physical activity research.

**Methods:**

In an interdisciplinary process, a framework was developed by experts for human rights policy and physical activity promotion. This framework is based on a conceptualization of the United Nation’s Committee on Economic, Social and Cultural Rights which defined – originally for the right to health – four attributes. These attributes were linked systematically to the field of physical activity promotion.

**Results:**

The framework is based on four attributes of PA as a human right: The availability, accessibility, acceptability and quality of PA programmes, facilities and services: (1) Availability includes, for example, the provision of exercise programs for vulnerable groups. (2) Accessibility has four overlapping dimensions: Nondiscrimination, physical accessibility, economic accessibility, and information accessibility. (3) Acceptability can include the sensitivity of a PA program for the culture of participating individuals and/or gender requirements. (4) And the quality of PA programmes, facilities and services, is, among other aspects, related to the pre- and in-service training of professionals.

**Conclusions:**

A framework based on the attributes of PA as a human right can be applied to promote PA among vulnerable groups. The framework can also help to improve physical activity among vulnerable groups such as, for instance, people with disabilities and women in difficult life situations.

**Funding:**

None

